# Insight into the genetic composition of South African Sanga cattle using SNP data from cattle breeds worldwide

**DOI:** 10.1186/s12711-016-0266-1

**Published:** 2016-11-15

**Authors:** Sithembile O. Makina, Lindsey K. Whitacre, Jared E. Decker, Jeremy F. Taylor, Michael D. MacNeil, Michiel M. Scholtz, Este van Marle-Köster, Farai C. Muchadeyi, Mahlako L. Makgahlela, Azwihangwisi Maiwashe

**Affiliations:** 1 Agricultural Research Council-Animal Production Institute, Private Bag X 2, Irene, 0062 South Africa; 2 Division of Animal Sciences, University of Missouri, Columbia, MO 65211 USA; 3 Department of Animal, Wildlife and Grassland Sciences, University of Free State, Bloemfontein, 9300 South Africa; 4 Delta G, Miles City, MT 59301 USA; 5 Department of Animal and Wildlife Sciences, University of Pretoria, Private Bag X 20, Hatfield, 0028 South Africa; 6 Agricultural Research Council-Biotechnology Platform, Private Bag X 5, Onderstepoort, 0110 South Africa

## Abstract

**Background:**

Understanding the history of cattle breeds is important because it provides the basis for developing appropriate selection and breed improvement programs. In this study, patterns of ancestry and admixture in Afrikaner, Nguni, Drakensberger and Bonsmara cattle of South Africa were investigated. We used 50 K single nucleotide polymorphism genotypes that were previously generated for the Afrikaner (n = 36), Nguni (n = 50), Drakensberger (n = 47) and Bonsmara (n = 44) breeds, and for 394 reference animals representing European taurine, African taurine, African zebu and *Bos indicus*.

**Results and discussion:**

Our findings support previous conclusions that Sanga cattle breeds are composites between African taurine and *Bos indicus.* Among these breeds, the Afrikaner breed has significantly diverged from its ancestral forebears, probably due to genetic drift and selection to meet breeding objectives of the breed society that enable registration. The Nguni, Drakensberger and Bonsmara breeds are admixed, perhaps unintentionally in the case of Nguni and Drakensberger, but certainly by design in the case of Bonsmara, which was developed through crossbreeding between the Afrikaner, Hereford and Shorthorn breeds.

**Conclusions:**

We established patterns of admixture and ancestry for South African Sanga cattle breeds, which provide a basis for developing appropriate strategies for their genetic improvement.

**Electronic supplementary material:**

The online version of this article (doi:10.1186/s12711-016-0266-1) contains supplementary material, which is available to authorized users.

## Background

South Africa is richly endowed with indigenous cattle breeds, among which are the Afrikaner, Nguni and Drakensberger breeds. These breeds played important roles in the social, cultural and economic development of the country [1]. Previously, Makina et al. [2] described these breeds as being genetically distinct from the European *Bos taurus* breeds (Angus and Holstein) and as having genomic regions associated with tropical adaptation [3]. Therefore, they may hold potential for production in harsh and fluctuating South African environments based on their adaptation to the nutritional, parasitic, and pathogenic challenges they are faced with. These breeds are not endangered and have reasonable effective population sizes [4–6]. Given their adaptive characteristics, they are potentially valuable to breeding programs in other regions that face biological stresses such as famine, drought or disease epidemics [7]. Furthermore, there is a worldwide drive for the effective management of indigenous genetic resources, which includes these breeds [7].

Afrikaner and Nguni cattle were brought to Southern Africa by the Khoi-Khoi people who migrated southwards from the African Great Lakes region between 600 and 700 AD [1]. Summers [8] postulated that ancestors of Afrikaner cattle migrated very quickly along the eastern side of Southern Africa to the current Western Cape and western parts of the Northern Cape. Ancestors of Nguni cattle are believed to have moved southward in the African continent at a much slower pace [8]. Afrikaner, Nguni and Drakensberger are classified as Sanga cattle and are thought to result from crossbreeding between thoracic-humped Lateral Horned zebu and humpless Egyptian Longhorn cattle [9–11]. The initial admixture probably occurred when African taurine cattle migrated south from Egypt and the Sudan, and indicine cattle migrated to the eastern seaboard of Africa from Arabia and India [9, 11]. However, Bisschop [12] suggested that Sanga cattle originated from crosses between humpless Egyptian Longhorn and short-horned *B. taurus brachyceros*. The cross-section of the horns of Egyptian Longhorn are oval, which is similar to those of Afrikaner cattle, while those of *B. taurus brachyceros* are round as in Nguni cattle [12].

Based on analyses with microsatellite markers, Hanotte et al. [11] and Freeman et al. [13] predicted that Sanga cattle resulted from the crossbreeding of African taurine and zebu cattle around 700 AD, which was confirmed by studies based on single nucleotide polymorphisms (SNPs) [14–18]. However, in spite of these studies, the genetic composition of South African Sanga cattle remains uncertain [19]. The genetic distance between cattle breeds appears, at least in part, inversely related to the geographic proximity of their origin [20]. Hanotte et al. [11] and Freeman et al. [13] also found that the extent of genetic introgression of zebu cattle across the African continent decreases from eastern to western Africa. MacHugh et al. [21] reported that the cattle breeds from the tsetse-infested areas of West and Central Africa had limited or no zebu ancestry, which concurs with their susceptibility to trypanosomiasis [21].

Genomic characterisation of South African Sanga cattle is a first step towards the development of appropriate breeding and selection strategies for these breeds. Makina et al. [2] characterized the relationships between the Afrikaner, Nguni, Drakensberger and Bonsmara breeds using Angus and Holstein as reference breeds, without including any other indicine or African taurine cattle. The limited number of breeds analyzed precluded detection of patterns of co-ancestry or admixture in the South African Sanga breeds. Thus, the aim of our study was to provide a more precise analysis of patterns of admixture and ancestry in the Afrikaner, Nguni, Drakensberger and Bonsmara cattle of South Africa using a subset of data that were generated for cattle breeds worldwide [14–17].

## Methods

### Description of samples and quality control

Genotypes for four South African Sanga cattle breeds [Afrikaner—AFR (n = 36), Nguni—NGU (n = 50), Drakensberger—DRA (n = 47) and Bonsmara—BON (n = 44)] originated from previous studies [2, 3, 6]. They were generated using the Illumina BovineSNP50 BeadChip v2, which features 54,609 SNPs distributed across the bovine genome with an average spacing of 49.9 kb [16]. These data were combined with genotypes from an additional 394 reference animals representing European taurine cattle i.e. Shorthorn (SH), Hereford (HFD), Simmental (SM), Limousin (LM), Angus (AN) and Holstein (HOL), African taurines i.e. N’Dama (NDAM), Somba (SOM), Kuri (KUR), Lagune (LAG) and Baoule (BAO), African zebu i.e. Ankole-Watusi (ANKW), Boran (BOR) and Sheko (SHK), East African zebu i.e. short-horned zebu (ZEB) and zebu Bororo (ZBO), and *Bos indicus* i.e. Brahman (BR), Nelore (NEL), Bhagnari (BAG) and Gir (GIR). Samples from these reference individuals were obtained with permission (see [22]) and were selected based on their land of origin and previous characterization. These samples originated from the following studies: Gautier et al. [14]; The Bovine HapMap Consortium [15]; Matukumalli et al. [16]; Decker et al. [17]; Gautier et al. [23]; and Decker et al. [24]. Additional file 1: Table S1 provides breed names and acronyms, number of individuals per breed, sampling area, land of origin and references to the original studies from which the samples came from.

These data were merged in PLINK [25] and autosomal SNPs that were common to all datasets were retained. This resulted in 35,155 SNPs and 548 individuals after removing SNPs with a MAF lower than 0.005, a call rate lower than 0.98 and individuals with more than 5% missing genotypes.

### Genetic relationships and population structure

Patterns of admixture and relationships among South African Sanga cattle in relation to the 20 reference breeds were determined using principal component analysis [26] implemented in the SNP Variation suite (SVS 8.1; Golden Helix Inc., Bozeman, Montana) and variational Bayesian inference as implemented in fastSTRUCTURE [27]. The data were evaluated for $K$ values ranging from 2 to 20 to evaluate ancestry proportions from $K$ ancestral populations assuming a simple non-informative prior. The $K_{\varepsilon }^{*}$ and $K_{\emptyset } c^{*}$ metrics from fastSTRUCTURE were used to determine the appropriate values of $K$ for the population structure explained by the dataset. The $K_{\varepsilon }^{*}$ metric is the value of *K*, which maximizes the log marginal likelihood lower bound and the $K_{\emptyset } c^{*}$ metric is the minimum value of $K$ that explains almost all of the ancestry in the dataset. Outputs from fastSTRUCTURE [27] were plotted using the GENESIS software [28]. To further test for evidence of admixture in South African Sanga cattle, ancestry graph [29], three-population ($f_{3}$) [30, 31] and four-population ($f_{4}$) tests [30, 32] implemented in TreeMix [29] were also used. The maximum likelihood tree (ancestry graph) [29] was first built for all 24 populations (see Additional file 2: Figure S1), after which, migration events were sequentially added to the tree until no more meaningful increases in the proportion of variance explained were observed (see Additional file 3: Table S2).

## Results

### Principal component analysis

**Fig. 1:**
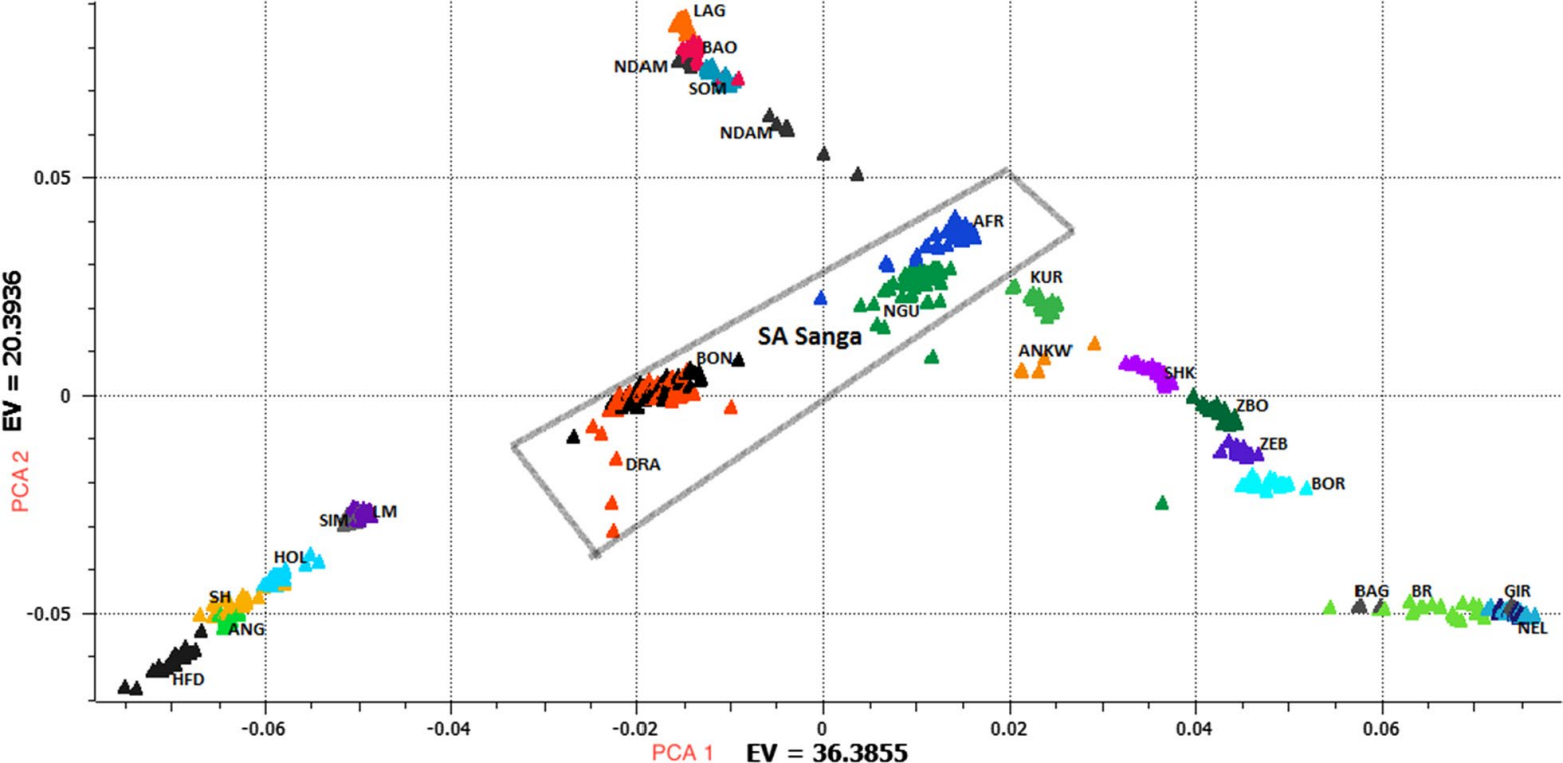
Principal component analysis incorporating South African Sanga breeds into a set of 20 worldwide cattle breeds. For full definitions of breeds (see Additional file 1 Table S1). *EV* explained variance

The principal component assessment agreed with previous findings, which partitioned bovine breeds into three distinct groups representing European taurines, African taurines and indicines [14–18, 23, 24] (Fig. 1). Afrikaner and Nguni cattle were situated on the gradient between the indicine and African taurine breeds, but more towards the latter. The Bonsmara and Drakensberger breeds clustered towards the centre of the triangle, which suggests that they have three ancestries (European taurine, African taurine and indicine).

### Population structure analysis

**Fig. 2:**
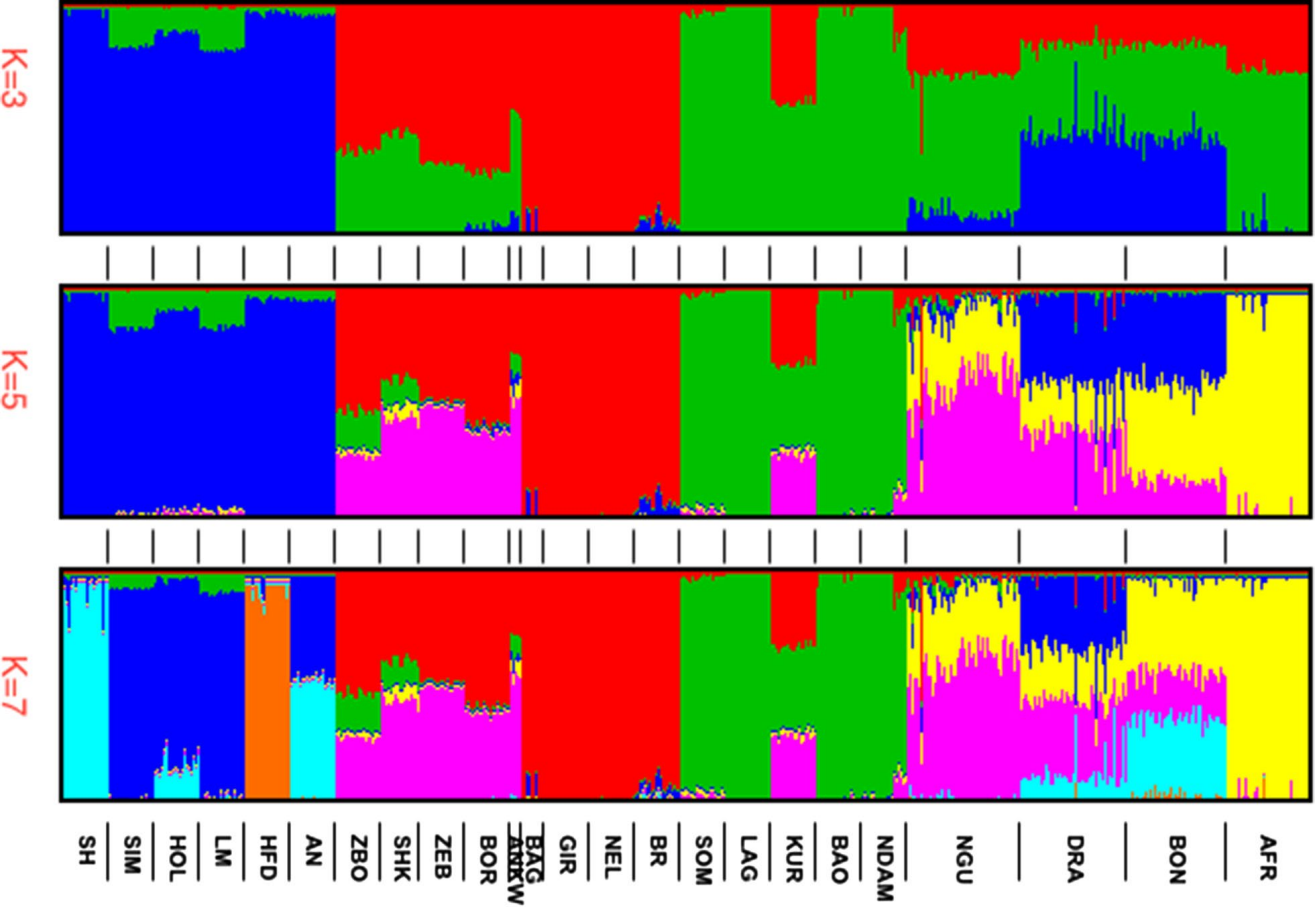
fastSTRUCTURE* bar plots* of proportions of genetic membership ($K = 3$–7). Each animal is represented by a vertical line divided into $K$ colours. Breed names are indicated at the bottom of the bar plots. For full definition of breeds (see Additional file 1: Table S1)

Allowing for three ancestral populations ($K = 3$) (Fig. 2) supported the classification of bovine populations into three distinct groups i.e. European taurine, African taurine and *Bos indicus*. This analysis predicted that the composition of Afrikaner cattle was approximately 70% African taurine and 28%, indicine, while that of Nguni was 60% African taurine, 30% indicine, and 10% European taurine. Predicted compositions of Bonsmara and Drakensberger were 41 and 46% European taurine, 42 and 38% African taurine, and 16 and 15% indicine, respectively.

Increasing $K$ from 3 to 5 assigned Afrikaner individuals into a single cluster and suggested that 97% of the Afrikaner genome was not shared with any of the reference breeds. The remaining 3% ancestral portion of the Afrikaner genome was shared with the African zebu breeds (1.6%) and with the African taurine and indicine reference breeds (<1%). Also at $K = 5$, Nguni, Drakensberger and Bonsmara remained admixed with a distinct genome component that was shared with African zebus (ZBO, ZEB, ANKW, SHK and BOR) and Kuri (a hybrid between African taurine and indicine populations [14]), but absent from indicines (BR, NEL, GIR, with the exception of BAG < 1%) and African taurines (NDAM, LAG, SOM and BAO). We note that the distinct component in BAG was only observed in a few individuals, which suggests that it may have been introduced through unsupervised crossbreeding.

Increasing $K$ to 7 separated European taurines into breed clusters and confirmed that the genetic composition of Bonsmara included Afrikaner (40%), Shorthorn (33%), African zebu (19%) and Hereford (5%). Drakensberger shared ancestry with African zebu (31%), Holstein (32%), Afrikaner (20%), and Shorthorn, (13%). Nguni was predicted to be a hybrid between African zebu (60%) and Afrikaner (30%), with traces of indicine (3%), European taurine (3%) and African taurine (2%) ancestry. Increasing $K$ further towards the number of populations studied assigned Drakensberger ($K = 9$) and Nguni ($K = 11$) to discrete clusters.

### Ancestry graph

**Fig. 3:**
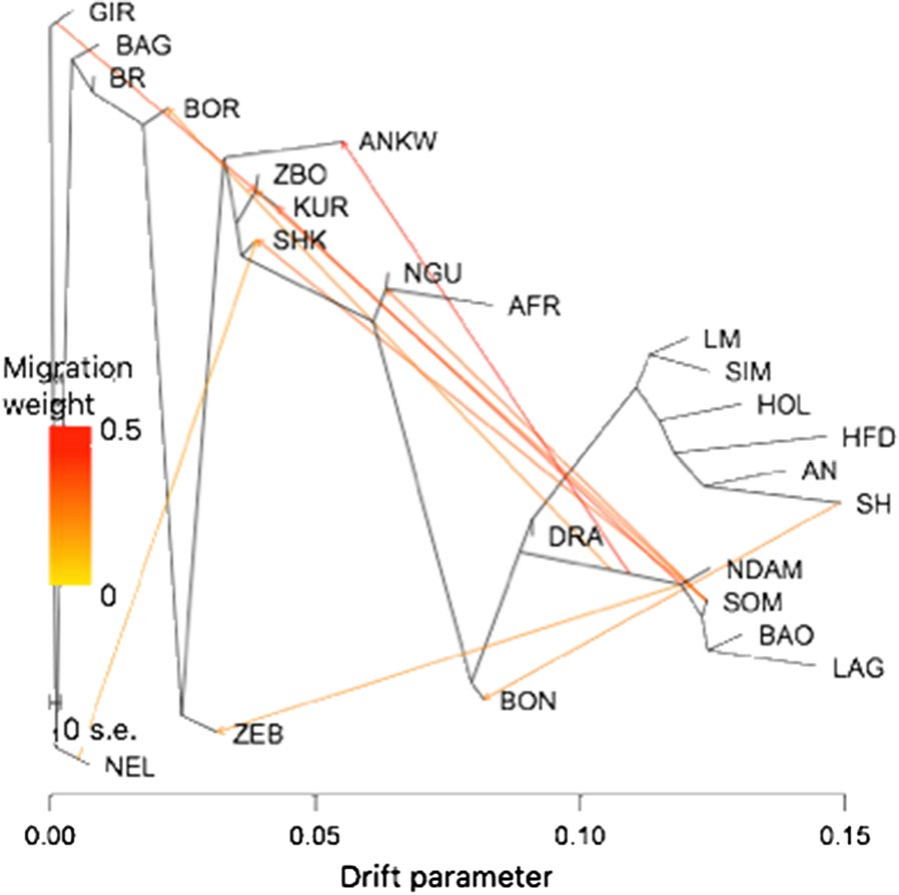
Graph of the inferred relationships between 20 cattle breeds from the worldwide subset and the South African Sanga cattle allowing for 10 migration edges. For full definition of breeds (see Additional file 1: Table S1]

The ancestry graph with 10 migration edges as developed using TreeMix [29] is in Fig. 3. This graph revealed the introduction of NDAM or NDAM relatives into the Nguni and Afrikaner cattle. This finding agrees with the results from the cluster analyses ($K = 3$), which indicated that the Afrikaner and Nguni cattle received approximately 60 to 70% of their ancestry from African taurine cattle (Fig. 2). In addition, we observed an admixture edge between Shorthorn and Bonsmara that was consistent with the history of the development of this breed. The eight other admixture processes modelled by network edges were previously characterized by Gautier et al. [14]; The Bovine HapMap Consortium [15]; Matukumalli et al. [16]; Decker et al. [17]; Gautier et al. [23]; and Decker et al. [24].

### Formal tests of admixture

The three-population statistic $f_{3}$(A;B,C) [30, 31] tests for bifurcating tree-like relationships in the evolution of populations and significant negative values of the $f_{3}$ statistic imply that population A is admixed and is a mixture of populations related to B and C [30, 31]. In agreement with results from the cluster analyses, we detected strong evidence of admixture in Drakensberger, Bonsmara and Nguni cattle. Examining Nguni in conjunction with any of the populations related to African taurines, indicines, African zebus, European taurines or Afrikaner yielded significant tests, for example, $f_{3}$[NGU;NEL,LAG] (Z-score = −18.00); $f_{3}$[NGU;BOR,LAG] (Z-score = −7.15); $f_{3}$[NGU;AN;AFR] (Z-score = −2.81) and $f_{3}$[NGU;SIM,AFR] (Z-score = −2.11). Similarly, significant negative values were detected for $f_{3}$ statistics for trios of $f_{3}$[BON;AFR,SH] (Z-score = −26.89) and $f_{3}$[BON;HFD,AFR] (Z-score = −14.93). The three population test for Drakensberger revealed that the most significant Z-scores were $f_{3}$[DRA;AFR,SH] (Z-score = −15.28); $f_{3}$[DRA;HOL,AFR] (Z-score = −12.98) and $f_{3}$[DRA;ANG,AFR] (Z-score = −12.02). The $f_{3}$ statistic failed to detect admixture within the Afrikaner cattle and agreed with the cluster analyses at $K = 5$ as described above.

The four population test $f_{4}$(A,B;C,D) [30, 32] tests whether (A, B) and (D, C) represent distinct clades in a population tree. In this test, significant non-zero values indicate the presence of gene flow between the (A, B) and (C, D) groups in the tree [30, 32]. In this test, combining Afrikaner and any of the African zebu or African taurine breeds yielded the most significant values (see Additional file 4: Table S3), which suggested gene flow from African zebu breeds or African taurines into the Afrikaner cattle. For Nguni, Bonsmara and Drakensberger, the most significant non-zero values were obtained when they were combined with any of the indicine, European taurine, African taurine and African zebu breeds (see Additional file 4: Table S3), which indicates the presence of gene flow between these breeds.

## Discussion

This study aimed at unravelling patterns of ancestry and admixture in South African Sanga cattle. Cluster analysis ($K = 3$) and the principal component analysis were mutually supportive and highlighted the presence of three genetic backgrounds for the populations studied. The classification of the bovine populations into a triangle-like structure is well-established [14–18, 23, 24]. As already mentioned, the Nguni and Afrikaner cattle were observed on the gradient between the indicines and African taurines, but more towards the African taurines, which indicates that the genomes of these breeds include more African taurine genetic background than indicine genetic background. This was supported by the proportions inferred by the admixture analysis at $K = 3$, which indicated that about 70 and 60% of the Afrikaner and Nguni ancestries were derived from African taurines. The detection of a migration edge from NDAM or NDAM relatives into Nguni and Afrikaner cattle in the TreeMix analysis provided further support. This larger proportion of ancestry shared between African taurines and South African Sanga (Afrikaner and Nguni) at $K = 3$ was consistent with the hypothesis of selection against susceptibility to trypanosomiasis, which may have led to a reduction in the extent of indicine ancestry in the surviving cattle, since indicine cattle are susceptible to trypanosomiasis [21, 33, 34].

As expected, Bonsmara and Drakensberger clustered towards the centre of the triangle in the principal component analysis, which suggests that these breeds have three ancestries (European taurine, African taurine and indicine). This was supported by the results of the cluster analysis at $K = 3$, which indicated that the Bonsmara and Drakensberger breeds were 41 and 46% European taurine, 42 and 38% African taurine, and 16 and 15% indicine, respectively. This was further confirmed by the detection of strong evidence for admixture by the three and four population tests ($f_{3}$ [30, 31] and $f_{4}$ [30, 32]) when these breeds were examined in conjunction with European taurines, African taurines and indicines. These results are consistent with the histories of the development of these breeds. The Bonsmara breed was developed at the Mara and Messina research stations between 1937 and 1963 under the guidance of the late Professor Jan C. Bonsma [35]. The aim was to produce a local breed that was well adapted to sub-tropical environments and had superior production compared to Afrikaner cattle. Five types of crosses were tested with Afrikaner cattle that included Red Aberdeen Angus, Hereford, Red Poll, Shorthorn and Sussex breeds. Pilot trials revealed that breed compositions including 5/8 Afrikaner and 3/8 Hereford or Shorthorn ancestries resulted in increased calving percentages and weaning weights with reduced calf mortalities relative to the purebreds [36]. Introgression of European taurine into the Drakensberger breed could have occurred due to the association of this breed with European settlers [1]. In 1837, several “Voortrekker” families (settlers) left Cape Province and traveled north with herds of similar black oxen that were then called “Vaderland” cattle. Most of these trekkers settled along the Drakensberg Mountain range and among them, the Uys family is believed to have played a significant role in the development of the “Uys-cattle” through strong selection within their closed herd; the “Uys-cattle” was later referred to as the Drakensberger breed by the Department of Agriculture owing to their prevalence in the pastures of the Drakensberg Mountains [36].

Allowing for five ancestral populations partitioned the African taurine and indicine admixture ancestry that was observed in the Nguni, Drakensberger and Bonsmara breeds ($K = 3$). With the exception of the African zebus (ZBO, ZEB, ANKW, SHK and BOR) and the Kuri (hybrid between African taurine and indicine populations [14]), this component was unique to the Nguni, Drakensberger and Bonsmara breeds and absent in indicine (BR, NEL, GIR) and African taurine (NDAM, LAG, SOM and BAO) cattle. Thus, the indicine component of African zebu and Sanga cattle seems to differ from that observed in other modern indicine cattle (NEL, BR and GIR). We hypothesize that strong selection within the African context caused the significant divergence between the indicine genome present in African admixed cattle and the genome of other modern indicine cattle. Alternatively, the founders of indicine cattle that migrated into Africa may have differed from those of the modern indicines used in the analysis.

Afrikaner cattle appear to have diverged from their ancestral populations and are recognized as a distinct breed ($K \ge 5$), which is likely due to the effects of genetic drift after admixture and strong selection of animals to conform to the standards and breeding objectives of the breed society. Afrikaner is the oldest indigenous cattle breed in South Africa and was the first indigenous breed to form a breed society in 1902 [1]. These results are consistent with the higher levels of inbreeding postulated by Coetzer and Van Marle [37] and detected by Makina et al. [2].

In agreement with the $f_{3}$ statistics, the Nguni cattle were predicted to be admixed ($K = 5\;{\text{and}}\;7$), their genetic makeup being predominantly African zebu with traces of indicines, African taurines and European taurines ancestries. The higher proportion of African zebu ancestry within the Nguni cattle is in agreement with the previous report by Makina et al. [3], who detected shared signatures of selection between Nguni and African zebu cattle. Historically, Nguni cattle were reared in extensive communal grazing systems in the presence of numerous other cattle representing various breeds and their crosses [38], which may also explain their admixture. The production potential of Nguni was only recognized in the early 1980s after the introduction of beef cattle recording schemes and the publication of results on the characterization of their productivity [39]. The Nguni breed society was established in 1986 and prior to this date, Nguni cattle were bred for various practical purposes and mated at random, which potentially led to admixture due to their close association with the indigenous people of South Africa and the communal husbandry they practiced [38].

In summary, our analyses support the view that Sanga cattle are composites of African taurine and *Bos indicus* [9, 11]. The Afrikaner breed clearly diverged from its ancestral forebears, probably due to genetic drift and alternative breeding objectives. The Nguni, Drakensberger and Bonsmara breeds are admixed, which was perhaps unintentional for Nguni and Drakensberger, but was certainly done by design in the case of Bonsmara that was developed through crossbreeding of Afrikaner, Hereford and Shorthorn.

## Conclusions

This study presents a comprehensive genome-wide characterization of South African Sanga cattle and confirms that South African Sanga cattle originated from African taurine and *Bos indicus*. The hybrid origin of Bonsmara cattle was confirmed and is consistent with the history of its development. Thus, genome-wide characterization of these populations has accurately recapitulated the history of the breeds’ formation [16]. These results improve our understanding of the composition and origins of South African Sanga cattle.

## Additional files

**Additional file 1: Table S1.** Description of samples included in the analyses. This table provides information about the samples used in this study such as the sample size, samples origin, land of origin and the references where the sample were previously characterized.

**Additional file 2: Figure S1** Graph of the inferred relationships between 24 cattle breeds without adding migration edges. For full definition of breeds see Additional file 1: Table S1. This graph shows the genetic relationship of South African cattle breeds in relation to the cattle breeds of the world before migration edges were sequentially added.

**Additional file 3: Table S2.** Explained covariance between populations for the ancestral graph with 10 migration edges. This data shows the changes in explained covariance between the populations as the number of migration edges added to the ancentral graph increases. It shows that after adding 10 migration edges, no significant changes were oberved in explained covariance.

**Additional file 4: Table S3.** Some significant $f_{4}$ statistics for South African Sanga cattle. This data shows some of the significant $f_{4}$ statistics and indicates the presence of gene flow between these breeds.

## References

[1] Scholtz MM (2010). Beef breeding in South Africa.

[2] Makina SO, Muchadeyi FC, van Marle-Köster E, MacNeil MD, Maiwashe A (2014). Genetic diversity and population structure among six cattle breeds in South Africa using a whole genome SNP panel. Front Genet.

[3] Makina SO, Muchadeyi FC, van Marle-Köster E, Taylor JF, Makgahlela L, Maiwashe A (2015). Genome wide scan for signatures of selection among six cattle breeds in South Africa. Genet Sel Evol.

[4] Tada O, Muchenje V, Dzama K (2013). Effective population size and inbreeding rate of indigenous Nguni cattle under in situ conservation in the low-input communal production system. S Afr J Anim Sci.

[5] Pienaar L, Neser FWC, Grobler JP, Scholtz MM, MacNeil MD (2015). Pedigree analysis of the Afrikaner cattle breed. Anim Genet Resour.

[6] Makina SO, Taylor JF, van Marle-Köster E, Muchadeyi FC, Makgahlela ML, MacNeil MD (2015). Extent of linkage disequilibrium and effective population size in four South African Sanga cattle breeds. Front Genet.

[7] Hanotte O, Jianlin H, Ruane J, Sonnino A (2005). Genetic characterization of livestock populations and its use in conservation decision making. The role of biotechnology in exploring and protecting agricultural genetic resources.

[8] Summers R (1960). Environment and culture in southern Rhodesia: a study in the “personality” of a land-locked country. Proc Am Philos Soc.

[9] Curson HH, Thornton RW (1936). A contribution to the study of African native cattle. Onderstepoort J Vet Sci Anim Ind..

[10] Epstein H (1971). The origin of the domestic animals of Africa. Vol. 1: dog, cattle, buffalo.

[11] Hanotte O, Bradley DG, Ochieng JW, Verjee Y, Hill EW, Rege JEO (2002). African pastoralism: genetic imprints of origins and migrations. Science.

[12] Bisschop JHR (1937). Parent stocks and derived types of African cattle, with particular reference to the importance of conformational characteristics in their study of origin. S Afr J Sci.

[13] Freeman AR, Bradley DG, Nagda S, Gibson JP, Hanotte O (2006). Combination of multiple microsatellite data sets to investigate genetic diversity and admixture of domestic cattle. Anim Genet.

[14] Gautier M, Flori L, Riebler A, Jaffrézic F, Laloé D, Gut I (2009). A whole genome Bayesian scan for adaptive genetic divergence in West African cattle. BMC Genomics.

[15] Gibbs RA, Taylor JF, Van Tassel CP, Barendse W, Eversole KA, Bovine HapMap Consortium (2009). Genome-wide survey of SNP variation uncovers the genetic structure of cattle breeds. Science.

[16] Matukumalli LK, Lawley CT, Schnabel RD (2009). Development and characterization of a high density SNP genotyping assay for cattle. PLoS One.

[17] Decker JE, Pires JC, Conant GC, McKay SD, Heaton MP, Chen K (2009). Resolving the evolution of extant and extinct ruminants with high-throughput phylogenomics. Proc Natl Acad Sci USA.

[18] Mbole-Kariuki MN, Sonstegard T, Orth A, Thumbi SM, Bronsvoort BM, Kiara H (2014). Genome-wide analysis reveals the ancient and recent admixture history of East African Shorthorn Zebu from Western Kenya. Heredity (Edinb).

[19] Scholtz MM, Gertenbach W, Hallowell G, Scholtz MM, Gertenbach W, Hallowell G (2011). History and origin of Nguni cattle. The Nguni breed of cattle: past, present, and future.

[20] McKay SD, Schnabel RD, Murdoch BM, Matukumalli LK, Aerts J, Coppieters W (2008). An assessment of population structure in eight breeds of cattle using a whole genome SNP panel. BMC Genet.

[21] MacHugh DE, Shriver MD, Loftus RT, Cunningham P, Bradley DG (1997). Microsatellite DNA variation and the evolution, domestication and phylogeography of taurine and Zebu cattle (*Bos taurus* and *Bos indicus*). Genetics.

[22] Decker JE, McKay SD, Rolf MM, Kim JW, Molina Alcalá A, Sonstegard TS, et al. Data from: Worldwide patterns of ancestry, divergence, and admixture in domesticated cattle. Dryad Digital Repository; 2014. doi:10.5061/dryad.th092. Accessed 20 Oct 2015. 10.1371/journal.pgen.1004254 PMC3967955 24675901

[23] Gautier M, Laloë D, Moazami-Goudarzi K (2010). Insights into the genetic history of French cattle from dense SNP data on 47 worldwide breeds. PLoS One.

[24] Decker JE, McKay SD, Rolf MM, Kim J, Molina Alcalá A, Sonstegard TS (2014). Worldwide patterns of ancestry, divergence, and admixture in domesticated cattle. PLoS Genet.

[25] Purcell S, Neale B, Todd-Brown K, Thomas L, Ferreira MA, Bender D (2007). PLINK: a tool set for whole-genome association and population-based linkage analyses. Am J Hum Genet.

[26] Patterson N, Price AL, Reich D (2006). Population structure and eigenanalysis. PLoS Genet.

[27] Raj A, Stephens M, Pritchard JK (2014). fastSTRUCTURE: variational inference of population structure in large SNP data sets. Genetics.

[28] Buchmann R, Hazelhurst S. Genesis manual. Technical Report 2015. University of the Witwatersrand. http://www.bioinf.wits.ac.za/software/genesis/Genesis.pdf.

[29] Pickrell JK, Pritchard JK (2012). Inference of population split and admixture from genome wide allele frequency data. PLoS Genet.

[30] Patterson N, Moorjani P, Luo Y, Mallick S, Rohland N, Zhan Y (2012). Ancient admixture in human history. Genetics.

[31] Reich D, Thangaraj K, Patterson N, Price AL, Singh L (2009). Reconstructing Indian population history. Nature.

[32] Keinan A, Mullikin JC, Patterson N, Reich D (2007). Measurement of the human allele frequency spectrum demonstrates greater genetic drift in East Asians than in Europeans. Nat Genet.

[33] Gifford-Gonzalez D, Hanotte O (2011). Domesticating animals in Africa: implications of genetic and archaeological findings. J World Prehist.

[34] Mwai O, Hanotte O, Kwon YJ, Cho S (2015). African indigenous cattle: unique genetic resources in a rapidly changing world. Asian Australas J Anim Sci.

[35] Bonsma JC (1980). Cross-breeding, breed creation and the genesis of the Bonsmara. Livestock production: a global approach.

[36] Dreyer CJ (1982). The breed structure of the Drakensberger cattle breed and factors that influence the efficiency of production.

[37] Coetzer WA, Van Marle J (1972). Die voorkoms van puberteit en daaropvolgende estrusperiode by vleisrasverse. S Afr J Anim Sci.

[38] Hofmeyr JH. Findings of the committee on a gene bank for livestock. In: Proceedings of the conference on conservation of early domesticated animals of southern Africa: 3–4 March 1994; Pretoria. 1994.

[39] Scholtz MM, Ramsey KA, Scholtz MM, Gertenbach W, Hallowell G (2011). Establishing a herd book for the Nguni breed in South Africa. The Nguni breed of cattle: past, present, and future.

