# Supplementary material for: Insight into the genetic composition of South African Sanga cattle using SNP data from cattle breeds worldwide
Source: Genet Sel Evol. 2016 Nov 15;48:88. doi: 10.1186/s12711-016-0266-1 (PMC5111355; doi:10.1186/s12711-016-0266-1)
Supplement: Supplementary file 1 — Additional file 1: Table S1. Description of samples included in the analyses. This table provides information about the samples used in this study such as the sample size, samples origin, land of origin and the references where the sample were previously characterized. [file 12711_2016_266_MOESM1_ESM.docx]

**Supplementary Materials**

Table S1: Description of samples included in the analyses

| **European taurine** | **Abbreviations** | **Sample size** | **Sample geographic origin** | **Land of origin** | **Reference for the data set** |
| --- | --- | --- | --- | --- | --- |
| Hereford | HFD | 20 | USA | Wales, Europe | [16, 24] |
| Limousin | LM | 20 | Massif Central, France | Europe | [16,24] |
| Beef Shorthorn | SH | 20 | England | Europe | [16,24] |
| Simmental | SIM | 20 | Bern, Switzerland | Europe | [16,24] |
| Angus | AN | 20 | USA | Aberdeenshire, Scotland | [16, 24] |
| Holstein | HOL | 20 | USA | Northern Europe | [16, 24] |
| **African taurine** |  |  |  |  |  |
| N’Dama | NDAM | 20 | Ivory Coast | Africa | [14, 15, 16, 17, 23, 24] |
| Somba | SOM | 20 | Boukombe (Benin) and Nadoba (Togo) | Africa | [14, 15, 16, 17, 23, 24] |
| Kuri | KUR | 20 | Lake Chad islands | Africa | [14, 15, 16, 23, 24] |
| Lagune | LAG | 20 | Porto Novo (Benin) | Africa | [14, 15, 16, 17, 23, 24] |
| Baoulé | BAO | 20 | Gaoua Ranch in Burkina-Faso | Africa | [14, 15, 16, 17, 23, 24] |
| **African Zebu** |  |  |  |  |  |
| Ankole-Watusi | ANKW | 5 | Ruanda | Africa | [15, 16, 23, 24] |
| Zebu bororo | ZBO | 20 | West-Africa | Africa | [14, 15, 16, 23, 24] |
| Boran | BOR | 20 | Southern Ethiopia | Africa | [14, 15, 16, 23, 24] |
| Sheko | SHK | 17 | East Africa | Africa | [14, 15, 16, 23, 24] |
| East African Shorthorn Zebu | ZEB | 20 | Kenya | Africa | [14, 15, 16, 17, 23, 24] |
| ***Bos indicus*** |  |  |  |  |  |
| Brahman | BR | 20 | USA | America, India | [14, 15, 16, 17, 23, 24] |
| Nelore | NEL | 20 | Brazil | America, India | [14, 15, 16, 17, 23, 24] |
| Bhagnari | BAG | 10 | Kaochi, Kalat, and Baluchistan, Pakistan | Asia | [14, 15, 16, 17, 23, 24] |
| Gir | GIR | 20 | Brazil | India | [14, 15, 16, 17, 23, 24] |
| **South African breeds** |  |  |  |  |  |
| Afrikaner | AFR | 36 | Free State Province, KwaZulu Natal | South Africa | [2, 3, 6] |
| Nguni | NGU | 50 | Gauteng, Limpopo, Northern Cape | South Africa | [2,3, 6] |
| Drakensberger | DRA | 47 | KwaZulu Natal, Mpumalanga | South Africa | [2, 3, 6] |
| Bonsmara | BON | 44 | Gauteng, Limpopo, Northern Cape | South Africa | [2, 3, 6] |
