# Supplementary figures and images for: Insight into the genetic composition of South African Sanga cattle using SNP data from cattle breeds worldwide
Source: Genet Sel Evol. 2016 Nov 15;48:88. doi: 10.1186/s12711-016-0266-1 (PMC5111355; doi:10.1186/s12711-016-0266-1)

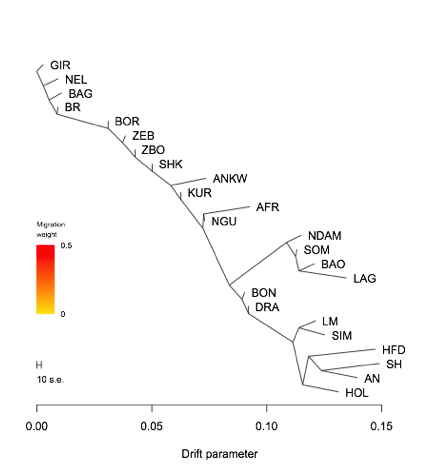

Supplement: Supplementary file 2 — Additional file 2: Figure S1 Graph of the inferred relationships between 24 cattle breeds without adding migration edges. For full definition of breeds see Additional file 1: Table S1. This graph shows the genetic relationship of South African cattle breeds in relation to the cattle breeds of the world before migration edges were sequentially added. [file 12711_2016_266_MOESM2_ESM.png]
