# Supplementary material for: Insight into the genetic composition of South African Sanga cattle using SNP data from cattle breeds worldwide
Source: Genet Sel Evol. 2016 Nov 15;48:88. doi: 10.1186/s12711-016-0266-1 (PMC5111355; doi:10.1186/s12711-016-0266-1)
Supplement: Supplementary file 3 — Additional file 3: Table S2. Explained covariance between populations for the ancestral graph with 10 migration edges. This data shows the changes in explained covariance between the populations as the number of migration edges added to the ancentral graph increases. It shows that after adding 10 migration edges, no significant changes were oberved in explained covariance. [file 12711_2016_266_MOESM3_ESM.docx]

Table S2: Explained covariance between populations for plotted ancestral graph with 10 migration edges

| Migration Edges | Explained variance |
| --- | --- |
| 0 | 0.960 |
| 1 | 0.967 |
| 2 | 0.972 |
| 3 | 0.975 |
| 4 | 0.979 |
| 5 | 0.981 |
| 6 | 0.984 |
| 7 | 0.985 |
| 8 | 0.988 |
| 9 | 0.990 |
| 10 | 0.993 |
| 11 | 0.992 |
| 12 | 0.993 |
