# Supplementary material for: Insight into the genetic composition of South African Sanga cattle using SNP data from cattle breeds worldwide
Source: Genet Sel Evol. 2016 Nov 15;48:88. doi: 10.1186/s12711-016-0266-1 (PMC5111355; doi:10.1186/s12711-016-0266-1)
Supplement: Supplementary file 4 — Additional file 4: Table S3. Some significant $f_{4}$f4 statistics for South African Sanga cattle. This data shows some of the significant $f_{4}$f4 statistics and indicates the presence of gene flow between these breeds. [file 12711_2016_266_MOESM4_ESM.docx]

Table S3: Some of the significant *f_4_* statistics for South African Sanga cattle.

| Breed |  |  |  | *f_4_* statistics | Std error | Z-score |
| --- | --- | --- | --- | --- | --- | --- |
| **Afrikaner** |  |  |  |  |  |  |
| AFR | BAG | ZEB | NDAM | -0.0448464 | 0.00116284 | -38.5661 |
| AFR | NDAM | ZEB | BAG | -0.00617416 | 0.000476035 | -36.5857 |
| AFR | ZEB | BAG | NDAM | -0.0386722 | 0.00119789 | -32.2836 |
| AFR | BAG | ZEB | LAG | -0.0472747 | 0.00129216 | -30.5688 |
| AFR | LAG | ZEB | BAG | -0.00733352 | 0.000510873 | -30.0341 |
| **Bonsmara** |  |  |  |  |  |  |
| BON | BOR | KUR | NGU | -0.0117377 | 0.000561838 | -61.0994 |
| BON | BAO | BOR | KUR | 0.0143496 | 0.000502674 | -56.984 |
| BON | SH | BOR | KUR | 0.00161571 | 0.000440801 | -47.7428 |
| BON | HFD | BOR | AFR | -0.00791495 | 0.000530583 | -35.4483 |
| **Drakensberger** | |  |  |  |  |  |
| DRA | BAO | HFD | SIM | 0.0039685 | 0.000350286 | -59.8206 |
| DRA | ZBO | BAO | HFD | -0.0155759 | 0.00068896 | -58.0636 |
| DRA | AN | BAO | HFD | 0.0234205 | 0.000705202 | -54.3042 |
| DRA | AFR | BAO | HFD | -0.0188942 | 0.00061046 | -53.0671 |
| **Nguni** |  |  |  |  |  |  |
| NGU | ANKW | BR | DRA | -0.0147372 | 0.00106494 | -61.3815 |
| NGU | BAO | BR | ANKW | 0.0129387 | 0.000701821 | -58.6298 |
| NGU | HFD | BR | ANKW | 0.00131526 | 0.000909563 | -57.4872 |
| NGU | AFR | BR | ANKW | 0.00124569 | 0.000384832 | -53.716 |
